# Polydopamine Particle as a Particulate Emulsifier

**DOI:** 10.3390/polym8030062

**Published:** 2016-02-26

**Authors:** Nobuaki Nishizawa, Ayaka Kawamura, Michinari Kohri, Yoshinobu Nakamura, Syuji Fujii

**Affiliations:** 1 Department of Applied Chemistry, Osaka Institute of Technology, 5-16-1 Omiya, Asahi-ku, Osaka 535-8585, Japan; m1m15509@st.oit.ac.jp (N.N.); yoshinobu.nakamura@oit.ac.jp (Y.N.); 2 Division of Applied Chemistry and Biotechnology, Graduate School of Engineering, Chiba University, 1-33 Yayoi-cho, Inage-ku, Chiba 263-8522, Japan; a.kawamura@chiba-u.jp (A.K.); kohri@faculty.chiba-u.jp (M.K.)

**Keywords:** Pickering emulsion, polydopamine, oil–water interface, crosslinking, colloidosome

## Abstract

“Pickering-type” emulsions were prepared using polydopamine (PDA) particles as a particulate emulsifier and *n*-dodecane, methyl myristate, toluene or dichloromethane as an oil phase. All the emulsions prepared were oil-in-water type and an increase of PDA particle concentration decreased oil droplet diameter. The PDA particles adsorbed to oil–water interface can be crosslinked using poly(ethylene imine) as a crosslinker, and the PDA particle-based colloidosomes were successfully fabricated. Scanning electron microscopy studies of the colloidosomes after removal of inner oil phase revealed a capsule morphology, which is strong evidence for the attachment of PDA particles at the oil–water interface thereby stabilizing the emulsion. The colloidosomes after removal of inner oil phase could retain their capsule morphology, even after sonication. On the other hand, the residues obtained after oil phase removal from the PDA particle-stabilized emulsion prepared in the absence of any crosslinker were broken into small fragments of PDA particle flocs after sonication.

## 1. Introduction

Emulsions stabilized with solid particles (so-called “Pickering emulsions”) have received great interest in the colloid and interface research area [1,2,3,4,5]. In essence, particulate emulsifiers offer more robust, reproducible formulations and lower toxicity profiles compared to conventional molecular-level emulsifiers. It has been well-known that various types of solid particles can work as emulsifier: inorganic particles such as silica [6,7], metals [8,9,10], semiconductors [11,12], clays [13], or ceramics [14,15]; organic particles such as bionano-particles including viruses [16] and proteins [17,18,19]; latex particles [20,21,22,23,24,25,26,27]; microgel particles [28,29,30,31]; and micelles [32] have been used as an effective particulate emulsifier.

Polydopamine (PDA), a mimetic of mussel adhesive proteins, has attracted much attention as a coating material without surface pre-treatments [33]. Dopamine monomer can be self-polymerized under basic condition on a variety of materials, such as metals, inorganic materials, and polymer materials. Extensive studies have been carried out to create PDA-coated materials with controllable film thickness and stability [34,35,36,37]. Some of the present authors have reported the preparation of PDA layers containing atom transfer radical polymerization initiating groups [38,39], polyethylene glycol moieties [40], dyes [41], and carboxylic acid-bearing compounds [42] to produce functional polymeric materials. Another advantage of PDA coating lies in their chemical structures that contain numerous functional groups, such as catechol and amine groups. Because of this advantage, PDA were easily modified by post-functionalization [43] or crosslinking [44]. Although most studies in which a PDA layer has been used have involved modifying the materials’ surface, there are a limited number of reports on the preparation of PDA in a form of particles, which were used for metal adsorbent materials [45], anti-cancer drug delivery [46], biomedical applications [47], and carbon source [48]. Unfortunately, the PDA particles synthesized previously are polydisperse in size, and synthesis of PDA particles with high monodispersity are desired. Under these situations, we have succeeded in fabrication of monodisperse PDA particles in water–methanol solution, and their use as bright structural color materials [49].

Herein, we describe the evaluation of PDA particle as a particulate emulsifier for the first time and utilize a liquid–liquid interface as a tool to assemble PDA particles. Thanks to catechol group on the PDA surface, PDA particles assembled at the droplet interface could be subsequently crosslinked using poly(ethylene imine) in order to stabilize these superstructures and to fabricate colloidosomes.

## 2. Materials and Methods

### 2.1. Materials

Reagents used to prepare PDA particles were dopamine hydrochloride (DA–HCl, Kanto Chemical, Tokyo, Japan), tris(hydroxymethyl)aminomethane (Tris, Kanto Chemical), and methanol (Kanto Chemical). Oils used to prepare emulsions were *n*-dodecane (≥99%, Sigma-Aldrich, Tokyo, Japan), toluene (99%, Sigma-Aldrich), dichloromethane (DCM, ≥99.0%, Sigma-Aldrich), methyl myristate (95.0%, Wako Pure Chemical, Osaka, Japan), octafluorotoluene (97%, Wako Pure Chemical) and perfluorononane (99%, Wako Pure Chemical). Poly(ethylene imine) (PEI, Average Molecular Weight, approximately 600, Wako Pure Chemical) was used as a crosslinking agent for PDA particles. Poly(vinyl alcohol) (PVA) were purchased from Sigma-Aldrich. Deionized water (<0.06 µS·cm^−1^, Advantec MFS RFD240NA: GA25A-0715) was used for preparation of PDA particles and emulsions. All other chemicals and solvents were of reagent grade and used as received.

### 2.2. PDA Particles Synthesis

DA–HCl (1.7 mg/mL, 10.8 mmol), Tris (14.4 g, 120 mmol), and water/methanol (*w*/*w* = 4/1) solution (1.2 L) were placed in a flask, and the mixture was stirred at 30 °C for 20 h. The PDA particles were separated and purified repeatedly by centrifugation (10,000 rpm (15,600× *g*) for 30 min) and redispersed in deionized water.

### 2.3. PDA Particles Characterization

#### 2.3.1. Scanning Electron Microscopy (SEM) Study

Scanning electron microscopy (SEM; JSM-6510A; JEOL, 20 kV, JEOL, Tokyo, Japan) studies were conducted on Pt sputter-coated (JFC-1600 Auto Fine Coater; JEOL) dried samples.

#### 2.3.2. Dynamic Light Scattering (DLS) and Zeta Potential Studies

The hydrodynamic diameter (*D*_h_; in water) and the zeta potential (in 0.01 M NaCl aqueous solution) of the PDA particles were measured by dynamic light scattering (DLS; ELSZ-1000ZS; Otsuka Electronics Co. Ltd., Osaka, Japan).

#### 2.3.3. Density

The solid-state density of the dried PDA particles was determined by helium pycnometry using a Micromeritics AccuPyc II 1340 instrument (Micromeritics, Norcross, GA, USA).

#### 2.3.4. Infrared (IR) study

IR spectra were measured by IR spectrophotometer (FTIR-420; JASCO, Tokyo, Japan).

### 2.4. Emulsion Preparation

Each volume (3.0 mL) of aqueous dispersion of the PDA particles with a solid concentration of 1.00 wt % and oil (*n*-dodecane, methyl myristate, toluene or dichloromethane) were placed in a glass vessel (inner volume, 13 mL). The two phases were homogenized for 2 min at 25 °C using a homogenizer (IKA ULTRA-TURRAX^®^ T 25 digital, Staufen, Germany) equipped with a dispersing element (S25N-8G: stainless steel, 18 mm stator diameter, 12.7 mm rotor diameter, 108 mm shaft length) operating at 20,000 rpm. Emulsion stabilities after standing for 24 h at 25 °C were assessed by gravimetric or visual inspection.

### 2.5. Crosslinking of Particle-Stabilized Emulsion

PEI aqueous solution (5.0 mL, 0.05–10 wt %) was added to the PDA particle-stabilized DCM-in-water emulsion (1.0 mL, prepared at a PDA particle concentration of 1.00 wt %) and magnetically stirred to ensure homogeneous mixing. The emulsion was then allowed to stand stirred at 25 °C for 1 h to allow colloidosome formation to occur.

### 2.6. Sonication Challenge

An aliquot of Pickering emulsion or colloidosome sample prepared using DCM (0.40 mL) was purified by five times replacement of supernatant with deionized water, and then was sonicated using an ultrasonic washing machine (Bransonic 221, Yamato Scientific Co., Tokyo, Japan) for 1 h. During the purification, DCM oil phase was removed from the droplets because of dissolution to repeatedly replaced water media. The sample was viewed by both optical microscopy and SEM.

### 2.7. Emulsion Characterization

#### 2.7.1. Drop Test

Emulsion type was confirmed using “drop test”. One drop of the emulsion was added to both water and oil and its ease of dispersion was assessed by visual inspection. Relatively rapid dispersion indicated that the continuous phase of the emulsion was the same as the diluent.

#### 2.7.2. Optical Microscopy (OM) Study

A drop of the diluted emulsion was placed on a microscope slide and viewed using an optical microscope (Motic BA200, Shimadzu, Kyoto, Japan) fitted with a digital system (Moticam 2000, Shimadzu).

#### 2.7.3. Laser Diffraction Study

A Malvern Mastersizer2000 instrument equipped with a small volume Hydro 2000 SM sample dispersion unit (*ca.* 150 mL including flow cell and tubing), a HeNe laser operating at 633 nm and solid-state blue laser source operating at 466 nm were used to size the emulsion droplets. The stirring rate was adjusted to 2000 rpm in order to avoid creaming of the emulsion. It was confirmed that droplet size did not change under these measurement conditions, which indicated no coalescence of the emulsion droplets. The raw data were analyzed using Malvern software. The mean droplet diameter was taken to be the volume average diameter (*D*_4/3_), which is mathematically expressed as *D*_4/3_ = Σ*D*_i_^4^*N*_i_/Σ*D*_i_^3^*N*_i_ (*D*_i_, the diameters of individual droplets; *N*_i_, the number of emulsion droplets corresponding to the diameters). The *D*_4/3_ values were shown plus–minus standard deviations, which were also determined using the Malvern software (Malvern Instruments, Malvern, UK). Droplet size can be measured from 0.02 to 2000 μm. Light diffraction method is an authorized technique to measure mean droplet diameters and their distributions. There is a high reproducibility in light diffraction measurements in our study.

#### 2.7.4. Scanning Electron Microscopy Study

Scanning electron microscopy (SEM; Keyence VE-8800, 12 kV, Keyence, Osaka, Japan) studies were conducted on Au sputter-coated (Elionix SC-701 Quick Coater, Sanyu Electron, Tokyo, Japan) dried samples.

## 3. Results and Discussion

### 3.1. PDA Particles Characterization

**Figure 1:**
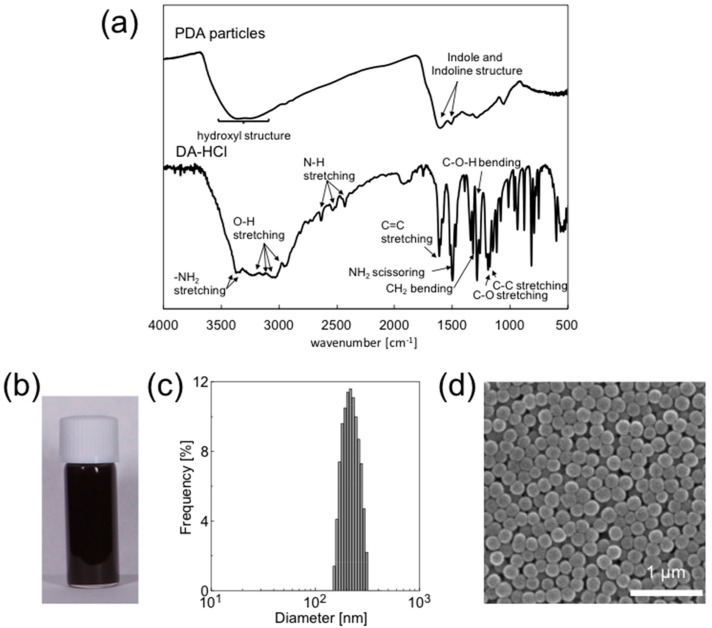
(**a**) FT-IR spectra of PDA particles and DA–HCl; (**b**) digital photograph of an aqueous dispersion of PDA particles (0.50 wt %); (**c**) size distribution of PDA particles measured by DLS; and (**d**) SEM image of PDA particles.

First, the PDA particles obtained were characterized by FT-IR spectroscopy (Figure 1a). The IR spectrum of the PDA particles shows a broad peak at 3200–3500 cm^−1^ due to the hydroxyl structures such as a catechol group. The characteristic peaks of indole and indoline structures at approximately 1600 cm^−1^ and approximately 1500 cm^−1^, respectively, were also found in PDA particles. Figure 1b shows a digital photograph of the PDA particle dispersion (0.5 wt % in water), which confirms the formation of black-colored aqueous dispersion. The hydrodynamic diameter, measured by DLS, was approximately 220 ± 39 nm (Figure 1c). The zeta potential of the PDA particles was measured to be approximately −42 mV (in 0.01 M NaCl aqueous solution), and the PDA particles with negative surface charges were well dispersed in water with no flocs were observed over a one-month period. The PDA particles size used in this study was measured to be 220 nm from the SEM image (Figure 1d), which accords well with that reported previously [49]. As indicated in the SEM image, PDA particles obtained are near monodisperse, which is consistent with the result obtained using DLS, and have smooth spherical surface. The PDA particles were used as a particulate emulsifier in their colloidally stable state.

### 3.2. Emulsions Stabilized with PDA Particles

#### 3.2.1. Different Oils Emulsion Data

In order to check the ability of the PDA particles as a Pickering-type emulsifier, four oils, namely *n*-dodecane, methyl myristate, toluene and DCM, were used as an oil phase to prepare emulsions at a PDA concentration of 1.00 wt %. *n*-Dodecane and methyl myristate are non-volatile at room temperature, and toluene and dichloromethane are volatile and used in order to characterize droplet after evaporation of oil phase in detail. In all cases, highly stable oil-in-water emulsions were achieved after homogenization and OM studies revealed that oil droplets stably dispersed in aqueous continuous phase without coalescence. All emulsions prepared in this study (beside methyl myristate) survived at least one month and nearly 100% emulsions remained in closed system where the evaporation of oil and water are not allowed: in the methyl myristate system, 39% demulsification occurred after two months.

Homogenization of the four oils in the absence of any PDA particles led to no/unstable emulsions: rapid macro-phase separation occurred. These results indicate that the PDA particles play an important role for the stabilization of the emulsions.

#### 3.2.2. Effects of PDA Particles Concentration on Emulsion Formation and Stability

It is worth asking whether the PDA particle concentration can be reduced below 1.00 wt % without affecting the emulsifier performance. Table 1 summarizes the results obtained by systematically reducing the PDA particle concentration from 1.00 to 0.05 wt % for *n*-dodecane as model oil. There is a clear trend of increasing droplet size with a decrease of PDA particle concentration. All the emulsions obtained were confirmed to be oil-in-water type from drop test although poor emulsions were obtained at or below PDA particles concentration of 0.05 wt %. The percentage of survived emulsion was calculated referring an equation (Equation (1)).

(1) $$Survived\;emulsion\;\%\;=\;100\;-\;\left(\frac{V_{oil}^{separated}}{V_{oil}^{initial}}\;\times \;100\right)\;$$

*V*_oil_^initial^: Initial oil volume prior to emulsification
*V*_oil_^separated^: Volume of separated oil

**Figure 2:**
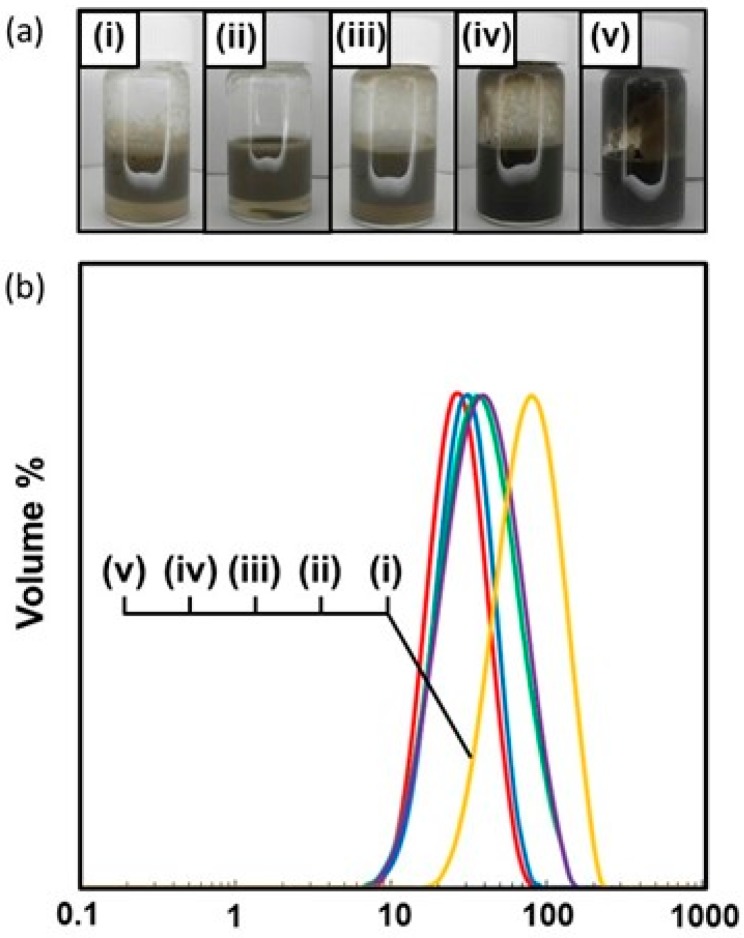
(**a**) Digital photographs; and (**b**) droplet size distribution curves of PDA-stabilized *n*-dodecane-in-water emulsions prepared at various PDA concentrations: (i) 0.05 wt %; (ii) 0.10 wt %; (iii) 0.20 wt %; (iv) 0.50 wt %; and (v) 1.00 wt %.

**Figure 3:**
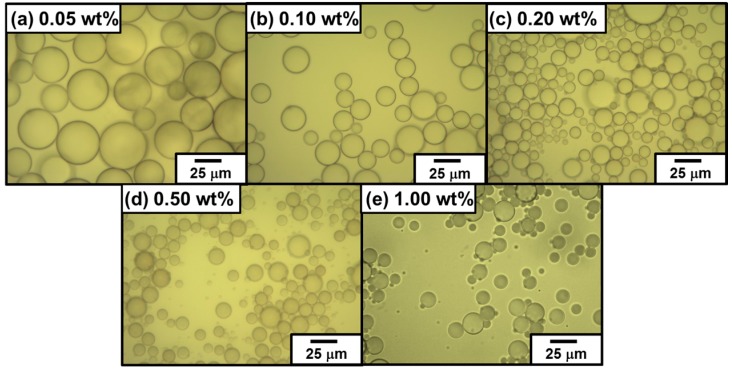
Optical micrographs of PDA-stabilized “Pickering-type” *n*-dodecane-in-water emulsions prepared at various PDA concentrations: (**a**) 0.05 wt %; (**b**) 0.10 wt %; (**c**) 0.20 wt %; (**d**) 0.50 wt %; and (**e**) 1.00 wt %. The emulsions were observed 24 h after preparation.

At and above the PDA particles concentration of 0.50 wt %, the emulsions were completely stable to coalescence over one week and only slow creaming was observed with time (see Figure 2a). At the PDA particle concentrations of 0.20 and 0.10 wt %, the emulsions were relatively stable: 100% emulsion survived for 24 h and only approximately 0.5% demulsification occurred after one week. At the PDA concentration of 0.05 wt %, the emulsion survived well (81% after one week), however demulsification occurred slowly to result in macrophase separation (only 66% emulsion survived) after 1.5 months. Typical OM images taken 24 h after emulsification in Figure 3 showed polydisperse oil droplets prepared at every PDA concentration. Number-average diameters were estimated using the optical micrographs as follows: 1.00 wt %, 11 ± 6 µm; 0.50 wt %, 12 ± 3 µm; 0.20 wt %, 13 ± 4 µm; 0.10 wt %, 17 ± 4 µm; and 0.05 wt %, 51 ± 14 µm (*n* =100). These number-average oil droplet diameters increased with a decrease of the PDA particle concentration, whose tendency accorded well with that estimated by the laser diffraction method (see Figure 2b and Table 1).

The fraction of PDA particles adsorbed at the oil–water interface can be readily estimated assuming a monolayer of adsorbed PDA particles is formed. Percentages of the PDA particles effectively attached on the oil–water interface based on the total amount of the PDA particles added were calculated using a following simple equation (Equation (2)) [17].

(2) $$\%\;PDA\;particles\;=\;\pi \;\frac{{R_{oil}}^{2}\;N_{oil}}{N_{part}\;{R_{part}}^{2}}\;\times \;100\;\%\;$$

where $N_{part}\;=\;\frac{W_{part}\;N_{A}}{Mw_{part}}\;$, and $N_{oil}\;=\;\frac{3\;V_{oil}}{4\;\pi \;{R_{oil}}^{3}}\;$.

Here, the calculations require some assumptions regarding the PDA particles packing efficiency at the interface, and the relatively polydisperse nature of the droplets can also introduce errors [13]. We assume 2D square lateral packing, uniform PDA particles and droplet sizes and PDA particle dimensions negligible as compared with those of oil droplets. We also assume that there are no PDA particles present in the oil phase because the energy barrier for the PDA particles to enter *n*-dodecane phase is too high. *R*_part_ and *R*_oil_ are the radii of the particles and oil, respectively (*R*_oil_ values used here were the volume mean radius determined by the laser diffraction method); *N*_part_ and *N*_oil_ are the numbers of particles and oil droplets, respectively; *V*_oil_ is the volume of oil; and *W*_part_ is the weight of PDA particles. The density of PDA (1.52 g/cm^3^) was used to calculate the PDA particle numbers used for preparation of emulsions. Laser diffraction studies (see Table 1) suggest mean volume-average droplet diameters of 30 ± 12, 33 ± 13, 44 ± 24, 46 ± 25 and 88 ± 40 µm obtained at the PDA particle concentrations of 1.00, 0.50, 0.20, 0.10 and 0.05 wt %, respectively. Within these constraints, the PDA particle adsorption efficiencies were estimated to be 352%, 682%, 1207%, 2307% and 1957%. This is a surprising result, and we need to re-examine the assumptions used for the calculations. The possible reason for these values over 100% efficiency should be due to non-closely packed PDA particles at oil–water interface in aqueous media at all the PDA particle concentrations. Recently, it has been reported that the PDA particles are expected to consist of electrostatically bonded PDA and DA oligomers [42,50,51], and there is a possibility that these DA oligomers can be dissolved into aqueous medium during storage after extensive centrifugal washing and could work as a molecular emulsifier. Actually, homogenization of the supernatant of the PDA particle aqueous dispersion, which was prepared by centrifugation, and *n*-dodecane led to formation of oil-in-water emulsion. From these results, it is expected that mixture of the PDA solid particles and DA oligomers eluting out from the PDA particles work as an emulsifier and the PDA particles are adsorbed to oil droplet surface in non-closely packed manner.

**Figure 4:**
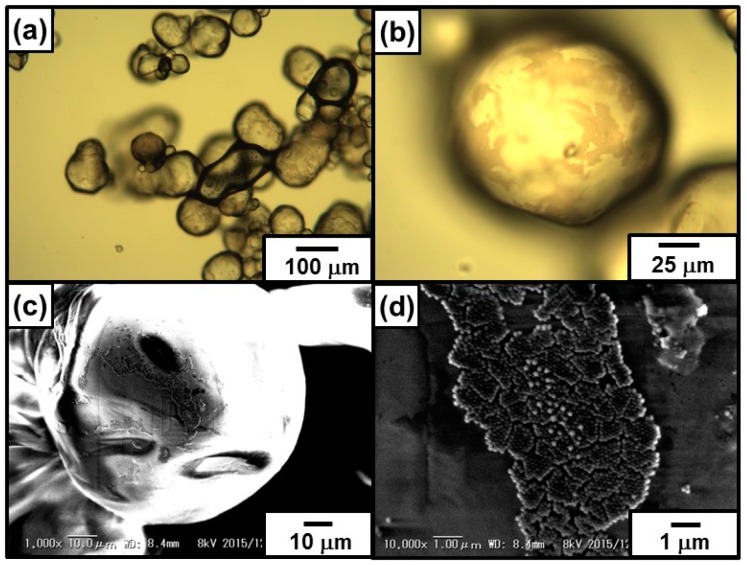
(**a**,**b**) Optical micrographs; and (**c**,**d**) SEM images of PDA-stabilized wax-in-water emulsion prepared using PDA particle aqueous dispersion (0.50 wt %). (**b**,**d**) are magnified images of (**a**,**c**), respectively. SEM images were taken without Au coating at an electron acceleration voltage of 8 kV.

In order to investigate the particle adsorption density at oil droplet surface in detail, wax was used as a solidifiable oil phase to stabilize oil-in-water emulsion. The particle-stabilized emulsion was successfully prepared using wax (3.0 mL) with a melting point of 58–60 °C by homogenization with an aqueous dispersion of PDA particles (3.0 mL, 0.50 wt %) at 70 °C. The prepared oil-in-water emulsion was cooled down to room temperature, and the liquid oil phase was solidified, which made possible for the droplets to be observed by SEM as well as OM (Figure 4). The droplet diameters were estimated to range from 10 to 200 µm (Heywood diameter, 123 ± 39 µm), and the PDA particles can be observed at droplet surface by OM (Figure 4a,b), thanks to their black color. Interestingly, the oil droplets were not fully covered with the PDA particles, and the PDA particles adsorbed at the surface formed islands. SEM studies also supported these OM results and magnified SEM images revealed the islands consisted of the PDA particle monolayer (Figure 4c,d). The area with no PDA particles should be stabilized with DA oligomers which cannot be observed using SEM due to their small sizes. There is a possibility that lateral capillary forces working between PDA particles at oil–water interface effectively attracted each other and the particles gathered to form islands [52]. Average percentage of the PDA particles at wax droplet surface was measured to be 42% ± 17%.

#### 3.2.3. Visualization of Transparent Emulsion

**Figure 5:**
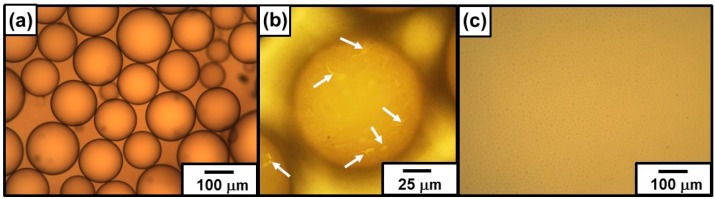
Optical micrographs of oil (mixture of 62% perfluorotoluene and 38 wt % perfluorononane)-in-water emulsion stabilized with (**a**,**b**) PDA particles and (**c**) PVA stabilizer. (**b**) is a magnified image of (**a**). The emulsion was prepared at 1.00 wt % stabilizer concentration and were observed 1 h after preparation. Arrows in (**b**) indicate bare oil–water interface that was not coated with the PDA particles.

PDA is known to be deeply colored and this color can be easily monitored with the naked eye. Therefore, PDA has been considered to be a good candidate as a coloring agent for creating patterned surface [53]. In the present study, the performance of the PDA particles as a colored particulate emulsifier was evaluated. Properties of the PDA material used in this study are its intense, intrinsic chromogenicity and its colloidal dimensions. A mixture of 62 wt % perfluorotoluene and 38 wt % perfluorononane, which has the same refractive index as water, was used as an oil phase. The refractive index of the oil and water was matched; therefore, in this emulsion system, light is not refracted or reflected by the oil–water interface, which generally leads to transparent emulsions. The PDA particles (1.00 wt %) proved to act as an effective particulate emulsifier for the oil, and the droplet test for the emulsion indicated that an oil-in-water emulsion was obtained. A typical OM image of the emulsion is shown in Figure 5a. The colored PDA particles attached to the oil–water interface for visualization of the emulsion droplets. The emulsion droplets were spherical and fairly polydisperse, with diameters ranging from 70 to 250 µm. Careful OM studies confirmed that there were bare oil–water interfaces that were not covered with the PDA particles on the droplet surface (Figure 5b), as already observed in wax-in-water emulsion system. In contrast, droplets could hardly be observed for the emulsion stabilized with PVA, which acts as a surface-active polymeric stabilizer (Figure 5c). The diameters of the droplets stabilized with PVA were between 5 and 20 µm and were smaller than those of the droplets stabilized with PDA particles. This is because molecular-level PVA emulsifier could stabilize larger oil–water interfacial area comparing with particulate PDA emulsifier.

#### 3.2.4. Emulsion Data with Dichloromethane as a Model Volatile Oil

**Figure 6:**
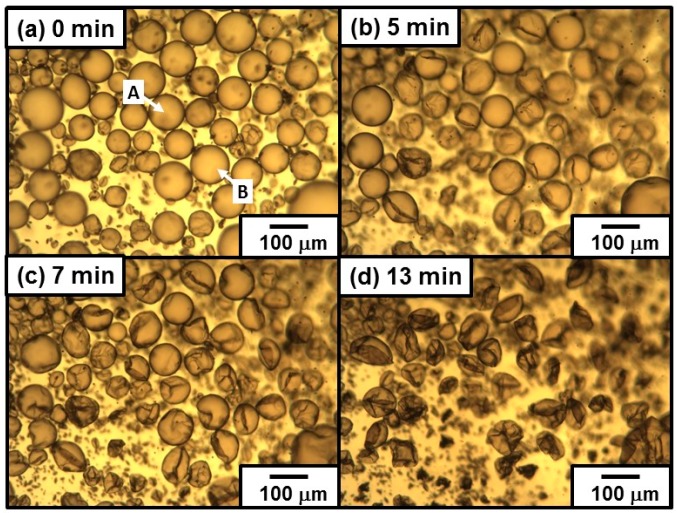
Optical micrographs illustrating evaporation of oil phase from DCM-in-water emulsion prepared using PDA aqueous dispersion (1.00 wt %). The emulsion was stored for 10 days after preparation before observation. The images were taken: (**a**) 0 min; (**b**) 5 min; (**c**) 7 min; and (**d**) 13 min after start of optical microscopy observation. The oil droplets indicated using arrows were used for estimation of circularity (see Figure 7).

**Figure 7:**
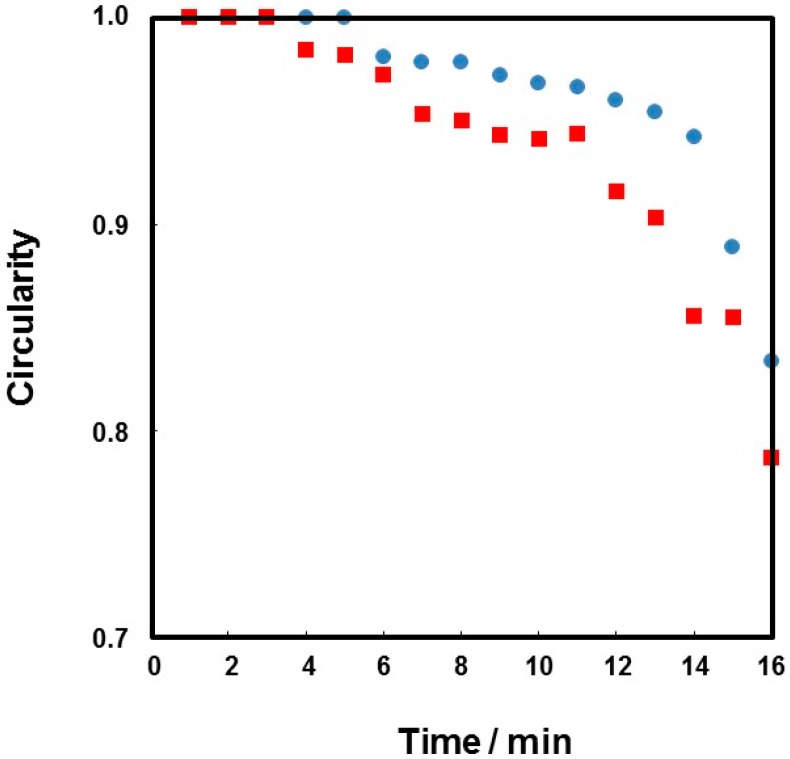
Relationship between time and circularity obtained for the oil droplets observed in Figure 6 (as indicated using arrows: ●, droplet A; ■, droplet B).

**Figure 8:**
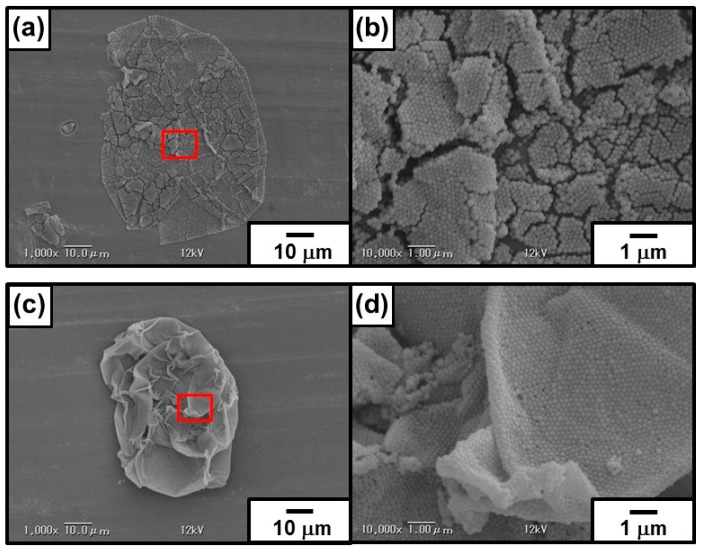
SEM images of “Pickering-type” DCM-in-water emulsion (**a**,**b**) without and (**c**,**d**) with crosslinking after the evaporation of DCM. (**b**,**d**) are magnified images of the areas shown in (**a**,**c**), respectively.

Recently, fabrication of colloidal assembly consisting of PDA particles has attained notable interest, because of their unique structural coloring character [49]. However, simple routes to direct and assemble PDA particles into shape-controlled constructs with hierarchical ordering are still lacking. We utilized the oil–water interface (that is, on the surface of oil droplets dispersed in continuous aqueous phase), which has been shown to be an ideal place for the assembly of colloidal particles, to fabricate capsule consisting of colloidal assembly shell. Homogenization of DCM and PDA aqueous dispersion (1.00 wt %) successfully led to stable DCM-in-water emulsion. OM studies recorded during the *in situ* evaporation of DCM gave important information of structure of the particle-stabilized oil droplets. The oil droplets, first, shrunk due to a decrease of oil volume remaining spherical shape. Then, slow droplet deformation from sphere to non-sphere occurred, and wrinkles appeared on the surface of droplet (see Figure 6). Relationship between time and circularity of oil droplets was shown in Figure 7, and it is clear that the circularity remained 1.0 until 4–5 min and then started to decrease. As indicated, the PDA particles were adsorbed to oil droplet surface in patchy manner, and it is expected that the droplet shrunk maintaining spherical shape until the PDA particles completely cover the droplet surface. After close-packed covering of the droplet surface with the PDA particles, the droplet shape started to deviate from sphere to non-sphere, because the volume of droplet decrease with keeping the fixed surface area. The appearance of the wrinkles is strong evidence for the attachment of PDA particles on the oil–water interface and stabilization of the emulsion. The SEM studies of the PDA residues remaining after evaporation of DCM and water from the emulsion revealed a wrinkled and ruptured capsule morphology (Figure 8a,b): this morphology accords well with that observed in the OM studies after the evaporation of DCM. The detailed SEM observation of the capsule confirmed the existence of cracks on the capsule surface, which should indicate there is no chemical bonding among the PDA particles and they attract each other via van der Waals force-based particle–particle interaction. Sonication of the PDA capsules dispersed in water medium led to breakage and formation of ill-defined PDA particle debris, which should indicate that the capsules were again formed via van der Waals force-based particle–particle interaction.

### 3.3. Formation of Colloidosomes from Particle-Stabilized Emulsion

In order to fabricate robust microcapsules, the PDA particles were connected with each other at oil–water interface of the droplets. These kinds of microcapsules are known as colloidosomes [20,23]. Covalent crosslinking is one of effective routes to connect the particles at oil–water interfaces, and some reactions (e.g., reactions between maleic anhydride and amine groups [54], epoxy and amine groups [55,56,57,58] and hydroxyl and isocyanate groups [59]) have been utilized. Here, the reaction between catechol and amine groups [44] was utilized to crosslink the PDA particles at droplet surfaces. Specifically, the PDA particles carrying catechol groups on their surfaces were crosslinked using water-soluble PEI crosslinker at oil–water interface. Although the contact angle of the PDA particles at oil–water interface is not known, the formation of an oil-in-water emulsion means that the contact angle must be less than 90° [3,4,5]. Thus, more than 50% surface area of each adsorbed particle should be exposed to the aqueous phase compared to the oil phase, which means large amount of the catechol groups on the particle surface must be available for reaction with the water-soluble PEI crosslinker.

**Figure 9:**
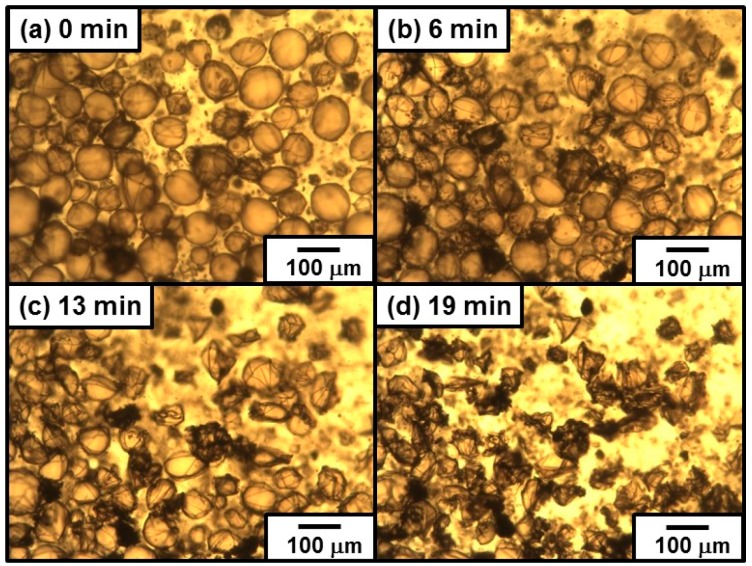
Optical micrographs illustrating evaporation of oil phase from colloidosomes prepared by crosslinking DCM-in-water emulsion shown in Figure 6. The images were taken: (**a**) 0 min; (**b**) 6 min; (**c**) 13 min; and (**d**) 19 min after start of optical microscopy observation.

Figure 9 shows OM images captured during evaporation of DCM from the colloidosomes prepared using 10 wt % PEI aqueous solution. The OM studies confirmed that the droplets had wrinkles on their surfaces from start of crosslinking reaction, rather than near-spherical shape observed in non-crosslinked precursor Pickering emulsion system. This difference should be due to partial removal of DCM oil phase from the droplets and decrease of the droplet surface area before crosslinking, which leads to close-packed PDA particles at the interface. Crosslinking reaction was conducted using the DCM-in-water emulsion with 10 days storage period at room temperature after preparation, and it is expected that small amount of DCM evaporated even closed with a lid, which should lead to near-closed packing of the PDA particles at droplet surface. The emulsion was diluted with PEI aqueous solution when the crosslinking reaction was conducted, which should also decrease the surface area of the droplets and close packing of PDA particles occur because of partial dissolution of DCM from the droplets into continuous aqueous phase.

**Figure 10:**
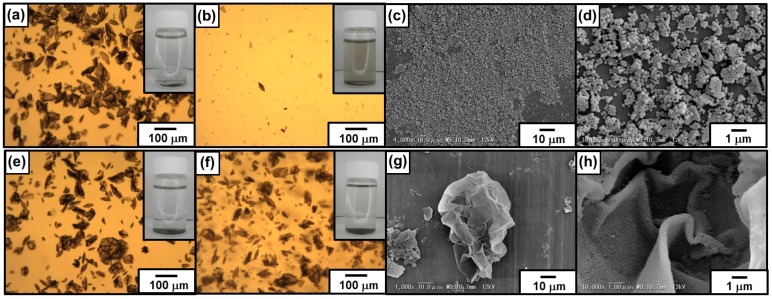
(**a**,**b**,**e**,**f**) Optical micrographs and (**c**,**d**,**g**,**h**) SEM images of PDA particle-stabilized DCM-in-water emulsions (**a**–**d**) without and (**e**–**h**) with crosslinking using 10 wt % PEI aqueous solution after removal of DCM oil phase. The emulsions were observed (**a**,**e**) before and (**b**–**d**,**f**–**h**) after sonication challenges. Insets shown in (**a**,**b**,**e**,**f**) are digital photographs of the samples.

Degree of circularity of colloidosome decreased with an increase of DCM evaporation time, as observed in precursor Pickering emulsion system. The SEM studies of the colloidosomes after evaporation of DCM and water revealed a wrinkled capsule morphology (Figure 8c,d). The microcapsule with wrinkles had few cracks on their surface, which should indicate there is crosslinking of the PDA particles successfully occurred. Successful crosslinking was also assessed by a sonication challenge, followed by OM and SEM observations. Sonication of the colloidosomes dispersed in aqueous medium after removal of DCM retained their microcapsule morphologies even after extensive sonication (Figure 10f–h), which should again indicate that the successful crosslinking occurred. On the other hand, the residues obtained after oil phase removal from the PDA particle-stabilized emulsion prepared in the absence of any crosslinker were broken into small fragments of PDA particle flocs after sonication (Figure 10b–d).

**Figure 11:**
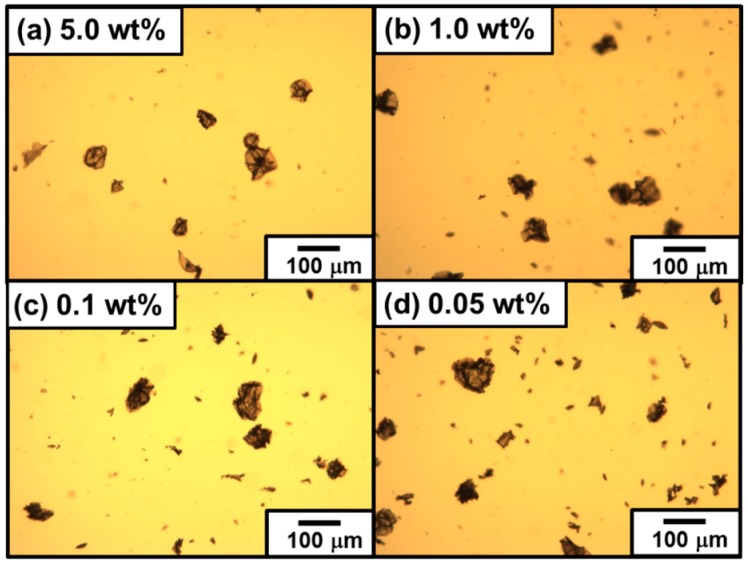
Optical micrographs of PDA particle-stabilized DCM-in-water emulsions after crosslinking using PEI aqueous solutions with various concentrations: (**a**) 5.0 wt %; (**b**) 1.0 wt %; (**c**) 0.1 wt %; and (**d**) 0.05 wt % PEI aqueous solution systems. The emulsions after removal of DCM oil phase were observed after sonication challenges.

It is worth investigating how much the PEI crosslinker concentration can be reduced without affecting colloidosome formation (Figure 11). OM studies on colloidosomes after extensive sonication challenge revealed that wrinkled microcapsules were main product for 5 wt % PEI solution system. At and below 1 wt % PEI solution systems, the broken capsules were observed and the amount of ill-defined PDA particle debris increased with a decrease of PEI concentration. These results indicated that the robustness of colloidosomes can be controlled by changing the crosslinking degree.

## 4. Conclusions

In summary, we described the first use of PDA as a particulate emulsifier. The PDA particles proved to be an effective Pickering emulsifier for the stabilization of oil-in-water emulsions. These emulsions were characterized in terms of their mean droplet diameter and morphology using laser diffraction, OM and SEM. SEM studies of the PDA residues remaining after evaporation of oil and water from the emulsion revealed a capsule morphology, which is strong evidence for the attachment of PDA particles on the oil–water interface and stabilization of the emulsion. The PDA particles adsorbed to the oil–water interface can be covalently crosslinked using PEI to form colloidosomes, which can retain their microcapsule morphology against sonication challenge. The understanding and thereafter the control over the unique organizations of the particle assembly at fluid–fluid interfaces render the possibility to fabricate functional nanostructured materials with hierarchical orderings, like ultrathin particle membranes. Chemical functionalization of PDA can open further opportunities for their use in different applications. These new colloidosomes are expected to become a useful drug delivery carrier, catalyst, and cosmetics.

## Figures and Tables

**Table 1:** Characterization data obtained for the emulsions prepared by adding PDA aqueous dispersion at various concentrations to *n*-dodecane. Equal volumes of oil and aqueous PDA dispersion were used and emulsification was carried out at 20,000 rpm for 2 min.

PDA Concentration/wt %	Type of Emulsion Formed	Survived Emulsion for 1 Week/ %	Volume-Average Oil Droplet Diameter/ μm
0.05	Oil/water	81	88 ± 40
0.10	Oil/water	99.5	46 ± 25
0.20	Oil/water	99.5	44 ± 24
0.50	Oil/water	~100	33 ± 13
1.00	Oil/water	~100	30 ± 12

## Abbreviations

PDA: Polydopamine
DA–HCl: Dopamine hydrochloride
DCM: Dichloromethane
PEI: Poly(ethylene imine)
PVA: Poly(vinyl alcohol)
SEM: Scanning electron microscopy
DLS: Dynamic light scattering
OM: Optical microscopy
